# Diversity in verbal fluency performance and its associations with MRI‐informed brain age matrices in normal ageing and neurocognitive disorders

**DOI:** 10.1111/cns.14144

**Published:** 2023-03-13

**Authors:** Hanna Lu, Jing Li, Ada Wai Tung Fung, Linda Chiu Wa Lam

**Affiliations:** ^1^ Department of Psychiatry The Chinese University of Hong Kong Hong Kong SAR China; ^2^ The Affiliated Brain Hospital of Guangzhou Medical University Guangzhou China; ^3^ Department of Applied Social Sciences The Hong Kong Polytechnic University Hong Kong SAR China

**Keywords:** brain age, cortical lateralization, gray matter volume, imaging, neurocognitive disorder, normal ageing, verbal fluency

## Abstract

**Introduction:**

Category verbal fluency test (CVFT) has been widely used to assess and monitor the cognitive capacities in epidemiological studies and clinical trials. Pronounced discrepancy in CVFT performance has been found in individuals with different cognitive statuses. This study aimed to combine the psychometric and morphometric approaches to decode the complex verbal fluency performance in senior adults with normal ageing and neurocognitive disorders.

**Methods:**

This study adopted a two‐stage cross‐sectional design involving quantitative analyses of neuropsychological and neuroimaging data. In study I, capacity‐ and speed‐based measures of CVFT were developed to evaluate the verbal fluency performance in normal ageing seniors (*n* = 261), those with mild cognitive impairment (*n* = 204), and those with dementia (*n* = 23) whose age range is from 65 to 85 years. In study II, structural magnetic resonance imaging‐informed gray matter volume (GMV) and brain age matrices were calculated in a subsample (*n* = 52) from Study I through surface‐based morphometry analysis. With age and gender as covariates, Pearson's correlation analysis was used to examine the associations of CVFT measures, GMV, and brain age matrices.

**Results:**

Speed‐based measures showed extensive and stronger associations with other cognitive functions than capacity‐based measures. The component‐specific CVFT measures showed shared and unique neural underpinnings with lateralized morphometric features. Moreover, the increased CVFT capacity was significantly correlated with younger brain age in mild neurocognitive disorder (NCD) patients.

**Conclusion:**

We found that the diversity of verbal fluency performance in normal ageing and NCD patients could be explained by a combination of memory, language, and executive abilities. The component‐specific measures and related lateralized morphometric correlates also highlight the underlying theoretical meaning of verbal fluency performance and its clinical utility in detecting and tracing the cognitive trajectory in individuals with accelerated ageing.

## Graphical abstract

The complex performance of category verbal fluency test (CVFT) could be decomposed into multiple components and examined in elderly with different cognitive statuses, including high‐performing, normal ageing, mild and major neurocognitive disorders (NCD) (a). Based on individual's magnetic resonance imaging (MRI) scans, cortical gray matter volume and brain age matrices can be computed and correlated with component‐specific CVFT proxies (b).

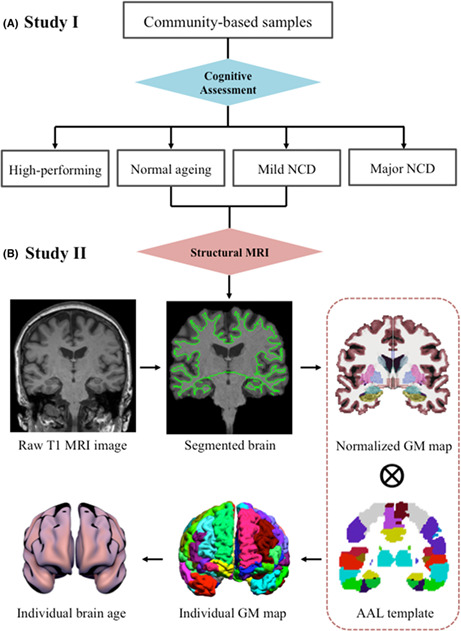

## 1 INTRODUCTION

As research on brain ageing moves forward, it is crucial to accurately evaluate the neurocognitive functions and develop simple and practical diagnostic biomarkers for individuals at early stage of cognitive impairments. Category verbal fluency test (CVFT), as one of the most efficient cognitive screening tools,^1^ is designed to evaluate the capacities to produce words under specific category within a fixed time interval (i.e., 30 or 60 s).^2^ During the past two decades, CVFT has been successfully used in the studies of healthy ageing^3^, ^4^ and different types of age‐related neurodegenerative diseases, including mild cognitive impairments (MCIs),^2^, ^5^, ^6^, ^7^ Alzheimer's disease (AD),^8^, ^9^, ^10^ frontotemporal dementia,^11^ Parkinson's disease (PD),^12^, ^13^ and dementia with Lewy bodies.^14^

Among these studies, the conventional interpretations of CVFT performance are often thought to be the measures of semantic verbal abilities and executive function.^15^, ^16^ However, given the nature of CVFT, the embedded qualities might not be adequately addressed by current interpretations. For instance, from a neuropsychological perspective, the raw scores of CVFT, including the numbers of correct named animals, fruits, and vegetables, are not a precise indicator of either verbal ability or executive function,^17^ but reflecting a pattern of diverse cognitive functions. The pattern's most important finding is that advanced age demonstrates differential effects on the domains of cognitive functions, which may be interpreted in two different ways: (1) Verbal abilities, referring to crystallized cognition, have a lower sensitivity to ageing process^18^; (2) Executive function and processing speed, as proxies of fluid cognition, keep deteriorating with normal and pathological ageing.^5^, ^19^, ^20^ Meanwhile, evidence from neuroimaging studies highlights the related neural underpinnings of verbal fluency performance, of which the patterns of brain–cognition correlations reflect the domain‐specific features, such as language ability, executive function.^4^, ^21^, ^22^, ^23^

Considering the differential age‐related effects on the verbal fluency performance, the clinical utilities of CVFT might be underestimated. First, several facets, such as executive control and the speed of word retrieval, implemented in the domain‐specific components of CVFT, are difficult to measure quantitatively. Second, the measures of CVFT performance demonstrate remarkable heterogeneity across a variety of populations, particularly in the patients with progressive cognitive decline.^24^, ^25^ The heterogeneity due to sample demographics and the diverse proxies of CVFT used across the studies may lead to inconsistent results and limit the clinical utilities of CVFT.

Taken together, to maximize the utilities of CVFT and develop component‐specific proxies for precise diagnostic and treatment evaluation, it may be helpful to practically dissociate the components of CVFT and achieve a thorough grasp of its neural underpinnings. In this study, we would first decode the CVFT performance into capacity‐based and speed‐based measures, and then investigate the component‐specific CVFT performance in elderly with different cognitive statuses. Second, we would quantify the cortical features and estimated brain age in a subsample and examine the relationships between brain features and component‐specific CVFT performance.

## 2 METHODS

### 2.1 Study I. Neuropsychology and psychometric matrices

#### 2.1.1 Participants

We recruited 488 community‐dwelling right‐handed old adults aged from 65 to 85 years from our previous cohort studies.^26^, ^27^ A structured neuropsychological battery was administered to evaluate the global cognition and the functions of core cognitive domains.^5^, ^28^ The Clinical dementia rating (CDR) scale, Cantonese version of Mini Mental State Examination (CMMSE), Alzheimer's Disease Assessment Scale‐Cognitive subscale, and Montreal Cognitive Assessment Hong Kong version (HK MoCA) were used to evaluate the global cognition and determined the cognitive statuses. The handedness was measured by the Edinburgh Handedness Inventory.^29^

Based on DSM‐5,^30^ the core domains of cognitive functions included the following: (1) Attention was measured by the digit span forward (DSF) and trail making test part A (TMT‐A); (2) Perceptual‐motor function was measured by the participant's performance on the commands as “tap each shoulder twice with two fingers keeping your eyes shut”; (3) Executive function was measured by TMT part B (TMT‐B); (4) Learning and memory were measured by the word list learning test, including immediate recall and delayed recall of the words, and working memory capacity.

Cerebrovascular risks were evaluated by the cumulative illness rating scale for geriatrics for the presence and severity of heart diseases, hyperlipidemia, diabetes mellitus, atrial fibrillation, hypertension, and anemia.^31^ The Pittsburgh sleep quality index,^32^ Cornell scale for depression in dementia,^33^ and activity of daily living scale^34^ were used to assess the subjective sleep quality, depressive symptoms, and everyday functioning separately. All the measurements were conducted with Chinese instructions.

The selection of high‐performing, normal ageing and neurocognitive disorders (NCDs) were based on their global cognitive performance measured by CMMSE. The details of criteria were as follows: (1) High performing: the ones in the top quartile of the normative scores in this cohort, presenting with CMMSE score greater than 29 and CDR equal to 0, were classified as high‐performing elderly. (2) Normal ageing: the ones with global cognitive function within 1.5 standard deviation (SD) of the age‐ and education‐adjusted normative scores, presenting with CMMSE score greater than 28 and CDR score equal to 0, were classified as normal ageing elderly. (3) NCD patients were determined by the following criteria: evidence of modest decline in one or more cognitive domains, which was set as ≥1.5 SD below the age‐ and education‐adjusted normative scores; no interference with independence in everyday activities; and no comorbid major psychiatric disorders. Mild NCD patients were defined as the CMMSE score less than 28 but greater than 22. Major NCD patients were diagnosed using a broad definition and met the following criteria: CMMSE score below the local cutoff for dementia of 18 and below for illiterate elderly, 20 and below for those with 1–2 years of education, and 22 and below for participants with more than 2 years of education.^35^

The exclusion criteria include the following: (1) past history of bipolar affective disorder or other psychosis; (2) history of major neurological disease, including stroke, brain tumor, transient ischemic attack, or traumatic brain injury; (3) comorbidities with severe sleep disorders and depressive symptoms; and (4) patients with PD and progressive supranuclear palsy.

#### 2.1.2 Evaluation of CVFT performance

On each trial, participants were required to overtly generate as many words as possible within 60 s. First, we conducted the CVFT by three trials: all participants were asked to produce the words in the categories of animal, fruit, and vegetable. Second, the number of the words produced in each category within 30 and 60 s was recorded. The proxies of CVFT performance used in this study were summarized as follows:

1. Capacity‐based measures: total number of correct words. As routine measures, sum of the words with all correct animals, fruits, and vegetables recorded in 30 and 60 s, excluding repetitions and intrusions are used as the raw scores of CVFT. Given the raw scores, the derived capacity score is calculated as the subtraction of the word number of CVFT 30 s from the word number of CVFT 60 s.
2. Speed‐based measures: speed of CVFT. The time that the participant produced a correct word of a predefined category within 30 and 60 s. The following formula:
  - Speed of CVFT 30 s (SOC30) = 30/number of corrected words in 30 s.
  - Speed of CVFT 60 s (SOC60) = 60/number of corrected words in 60 s.
  - Speed of CVFT capacity (SOC capacity) = 30/derived capacity score.

### 2.2 Study II. Neuroimaging and brain age matrices

#### 2.2.1 Participants

We randomly invited the eligible participants from Study I to participate the neuroimaging study. High‐resolution T1‐weighted magnetic resonance imaging (MRI) images of 52 elderly were acquired in the Prince of Wales Hospital using a 3.0 Tesla MRI scanner (Philips Healthcare). The acquisition of MRI data was configured with the following parameters: axial acquisition with a 256 × 256 × 192 matrix, no gap, thickness = 1 mm, field of view = 230 mm, repetition time = 2070 ms, echo time = 3.93 ms, flip angle = 15°. The sequence yielded high‐quality isotropic images with the voxel size of 1 mm × 1 mm × 1 mm.

#### 2.2.2 Surface‐based morphometry analysis

**FIGURE 1:**
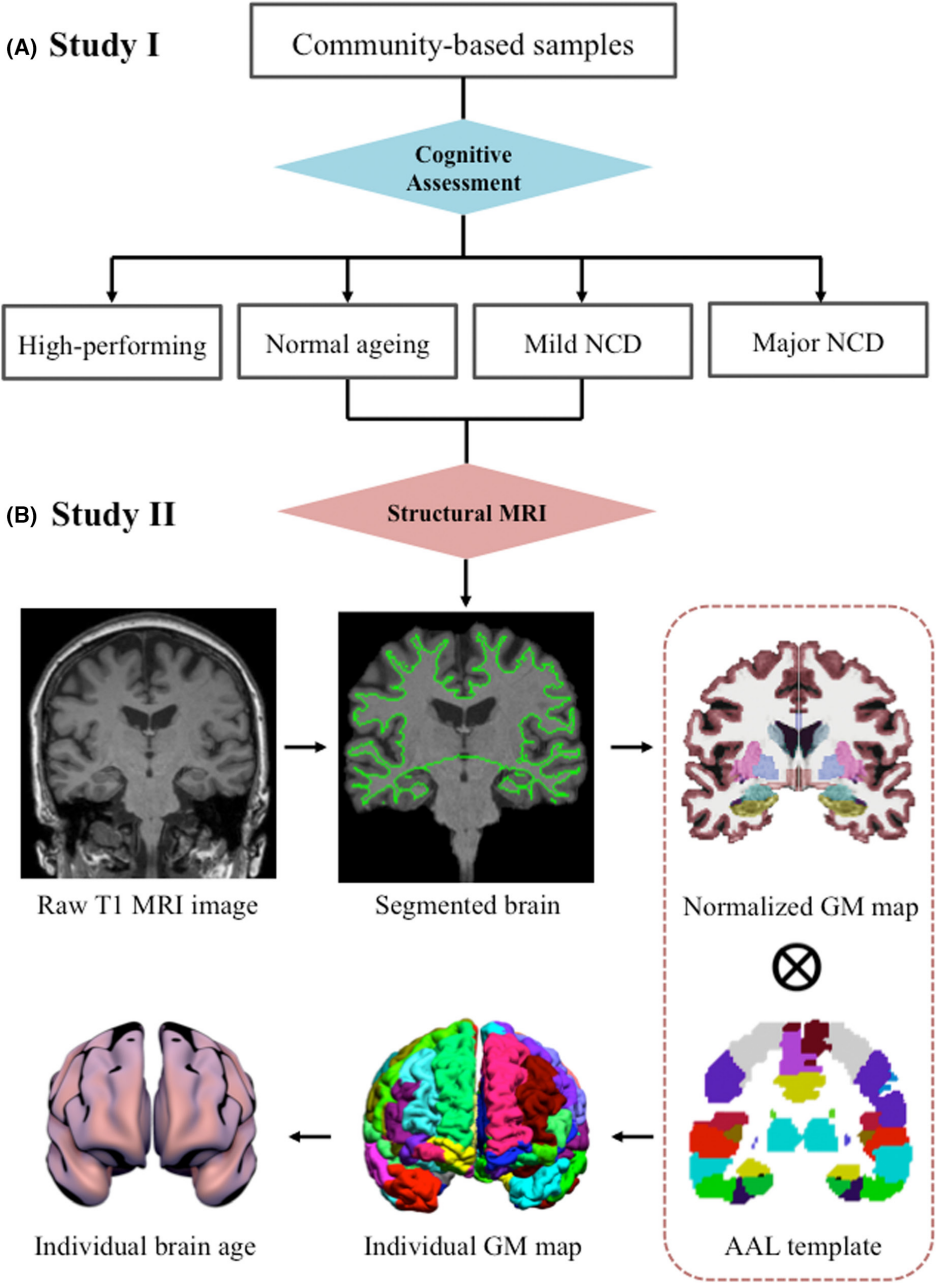
The flowchart of this study. (A) Study I: Psychometric mapping of category verbal fluency test (CVFT) in high‐performing, normal ageing, mild and major neurocognitive disorders (NCD); (B) Study II: Magnetic resonance imaging (MRI)‐informed gray matter volume mapping and brain age calculation. Abbreviations: AAL, Automated anatomical atlas; GM, Gray matter.

Brain morphometry including gray matter volume (GMV) was analyzed individually based on the Automated Anatomical Labeling (AAL) template using BrainSuite 16.0 (http://brainsuite.org/) with default parameters.^36^, ^37^ As shown in Figure 1, the detailed steps of data processing included the following: (1) Correction of head motion and segmentation of brain tissue; (2) Co‐registration of individual's MRI image to AAL template; and (3) Extraction of region‐specific GMV. Cortical lateralization was determined by laterality index (LI) calculated by the formula: LI = (left GMV − right GMV)/(left GMV + right GMV).^38^ LI > 0 indicates left lateralization; LI < 0 indicates right lateralization.

#### 2.2.3 Computation of brain age matrices

Pre‐trained brain age model contextualized the whole brain morphometric features of the training sets from the Cambridge Centre for Aging and Neuroscience project (Cam‐CAN) project (*N* = 611, age range: 18–90 years) (https://www.cam‐can.org) were first constructed to generate a machine learning‐based pre‐trained model.^39^ The brain age matrices were predicted using the support vector regression algorithm implemented in MATLAB (i.e., “fitrsvm” function, kernel: linear). Using 10‐fold cross‐validation, the estimated brain age model was applied to the entire samples (*N* = 611). Second, the participants from Study II were used as testing set (*N* = 52) for calculating and validating the brain age in clinical samples. The “Brain Age Gap Estimation” (BrainAGE) score is calculated as the difference between predicted brain age and chronological age, which indicates accelerated ageing process (positive value) or resilience (negative value).^40^, ^41^, ^42^

### 2.3 Data analysis

All data were tested for normal distribution through the Shapiro–Wilk test. Only data with a normal distribution could be counted. Homogeneity of variance test was used to evaluate the equality of variances among high‐performing, normal aging, minor NCD and major NCD groups. Group‐wise differences of demographics and neuropsychological performance were tested either with *χ* ^2^ test for category variable or one‐way analysis of variance (ANOVA) for continuous variables. Repeated measures ANOVA analyses were applied to evaluate changes from the numbers of corrected words of animal to vegetable. Two time points, including the 30 and 60 s, were treated as a within‐subject factor and the differences across four groups were treated as a between‐subjects factor. The interactions between time points and cognitive status were also examined in the above analyses. Covariates such as age, gender, and educational level were included in the model. Partial eta squared (*η* ^2^) was reported as effect size. Post‐hoc multiple comparisons among means were conducted using a Tukey HSD test when ANOVA results detected significant differences among the groups. We used Pearson correlation coefficient to test the relationships between CVFT measures, cognitive functions, and brain features. We carried out receiving operating characteristic (ROC) analysis to evaluate the power of CVFT measures in differentiating the seniors with different cognitive status. Significance levels were set at *p* value less than 0.05. Bonferroni correction was conducted to reduce the chances of obtaining false‐positive results of correlation analysis.^43^ ANOVA, *χ* ^2^ test, correlation analysis, and repeated measures ANOVA analyses were performed by IBM SPSS (Version 20).

## 3 RESULTS

### 3.1 Psychometric properties of CVFT

There were no differences in chronological age and gender ratio across the groups with different cognitive statuses. As shown in Table 1, mild and major NCD patients showed worse cognitive functions and lower daily activities than high‐performing and normal aging elderly. As to CVFT performance, the capacity‐based CVFT scores were coordinately getting lower with the severity of disease. Similarly, significant group‐wise discrepancy was also found in speed‐based measures of CVFT (Table 2). Repeated measures ANOVA analyses found significant interactions between time interval and cognitive status (*F* = 35.16, *p* < 0.001, *η* ^2^ = 0.18) on the CVFT performance. Moreover, post‐hoc analyses showed that the differences in component‐specific CVFT performance remained significant between two groups (Tables A1, A2, A3).

**TABLE 1:** Demographics and neurocognitive characteristics across four cognitive groups.

	High‐performing (*n* = 56)	Normal ageing (*n* = 205)	Minor NCD (*n* = 204)	Major NCD (*n* = 23)	*F* value	*p* Value
Age	69.27 ± 3.41	70.13 ± 4.11	71.04 ± 4.61	74.24 ± 3.75	3.09	0.065
Gender (M/F)	20/36	80/125	93/111	10/13	2.19	0.088
Education (years)	12.27 ± 3.98	9.82 ± 4.27	7.61 ± 4.26	4.65 ± 5.14	28.56	<0.001
CSDD	0.31 ± 0.97	0.54 ± 1.89	0.48 ± 1.42	2.26 ± 3.95	7.51	<0.001
PSQI	5.55 ± 3.22	5.71 ± 3.62	6.09 ± 3.49	7.22 ± 4.84	1.82	0.241
ADL	0.99 ± 0.01	0.99 ± 0.02	0.98 ± 0.03	0.86 ± 0.12	91.41	<0.001
CDR‐SOB	0.05 ± 0.14	0.19 ± 0.32	0.93 ± 0.63	3.33 ± 1.51	153.57	<0.001
CMMSE	29.55 ± 0.57	28.65 ± 1.05	27.04 ± 1.81	20.65 ± 2.85	238.67	<0.001
HK MoCA	29.25 ± 0.79	27.59 ± 1.45	23.96 ± 2.69	16.48 ± 4.21	278.09	<0.001
DSF	8.18 ± 0.94	7.66 ± 1.07	7.08 ± 1.21	6.22 ± 1.56	25.65	<0.001
DSB	4.79 ± 1.49	3.81 ± 1.31	3.11 ± 1.05	2.35 ± 1.53	37.51	<0.001
TMT‐A	11.09 ± 6.21	12.86 ± 6.27	17.42 ± 10.04	37.89 ± 23.25	54.85	<0.001
TMT‐B	45.17 ± 19.51	65.52 ± 42.91	93.37 ± 56.32	160.15 ± 52.58	32.81	<0.001

*Note*: Data are raw scores and presented as mean ± SD.

Abbreviations: ADL, Activity of daily living scale; CDR‐SOB, Clinical dementia rating‐sum of box; CSDD, The Cornell Scale for Depression in Dementia; DSB, Digit span backward; DSF, Digit span forward; HK MoCA, Montreal Cognitive Assessment Hong Kong version; NCD, Neurocognitive disorder; PSQI, Pittsburgh Sleep Quality Index; TMT‐A, Trail making test‐part A; TMT‐B, Trail making test‐part B.

**TABLE 2:** Comparisons of component‐specific CVFT performance across four cognitive groups.

	High‐performing (*n* = 56)	Normal ageing (*n* = 205)	Minor NCD (*n* = 204)	Major NCD (*n* = 23)	*F* value	*p* Value
Capacity‐based measures						
CVFT‐A30	14.71 ± 2.96	12.96 ± 2.91	11.07 ± 2.93	8.04 ± 3.07	43.59	<0.001
CVFT‐A60	20.73 ± 4.11	18.66 ± 4.35	15.31 ± 3.88	11.22 ± 3.59	53.22	<0.001
CVFT‐F30	12.14 ± 2.35	10.17 ± 2.22	8.91 ± 2.24	6.65 ± 1.89	48.58	<0.001
CVFT‐F60	16.21 ± 3.37	13.84 ± 2.95	11.69 ± 2.81	8.91 ± 2.91	56.12	<0.001
CVFT‐V30	11.93 ± 2.54	10.24 ± 2.61	8.95 ± 2.68	6.65 ± 2.66	32.06	<0.001
CVFT‐V60	18.29 ± 4.28	15.57 ± 4.04	12.88 ± 4.03	9.17 ± 3.03	45.79	<0.001
CVFT‐CA	6.02 ± 2.76	5.71 ± 2.81	4.19 ± 2.19	3.17 ± 1.83	19.45	<0.001
CVFT‐CF	4.07 ± 2.28	3.67 ± 1.84	2.79 ± 1.64	2.26 ± 1.68	14.05	<0.001
CVFT‐CV	6.36 ± 2.71	5.32 ± 2.44	3.93 ± 2.28	2.52 ± 1.21	27.62	<0.001
Speed‐based measures						
CVFT‐A30	2.12 ± 0.42	2.44 ± 0.64	2.89 ± 0.84	4.53 ± 2.36	52.97	<0.001
CVFT‐A60	3.01 ± 0.59	3.38 ± 0.77	4.21 ± 1.26	6.07 ± 2.63	58.55	<0.001
CVFT‐F30	2.56 ± 0.49	3.11 ± 0.73	3.63 ± 1.09	4.95 ± 1.74	45.79	<0.001
CVFT‐F60	3.87 ± 0.86	4.54 ± 1.04	5.46 ± 1.47	7.49 ± 2.67	56.61	<0.001
CVFT‐V30	2.65 ± 0.71	3.14 ± 0.92	3.71 ± 1.34	5.13 ± 1.75	33.99	<0.001
CVFT‐V60	3.47 ± 0.84	4.14 ± 1.18	5.16 ± 1.78	7.25 ± 2.34	50.43	<0.001
CVFT‐CA	6.43 ± 4.29	6.91 ± 4.83	9.61 ± 7.03	12.34 ± 6.73	12.62	<0.001
CVFT‐CF	10.59 ± 8.28	10.37 ± 6.82	12.84 ± 8.88	13.41 ± 9.36	3.91	0.009
CVFT‐CV	5.34 ± 2.35	7.47 ± 5.89	10.88 ± 8.18	13.31 ± 7.66	17.15	<0.001

*Note*: Data are raw scores and presented as mean ± SD.

Abbreviations: A30, Animal category at 30 s; A60, Animal category at 60 s; CVFT, Category Verbal fluency Test; CVFT‐CA, Category Verbal fluency test‐Capacity of Animal category; CVFT‐CF, Category Verbal fluency test‐Capacity of Fruit category; CVFT‐CV, Category Verbal fluency test‐Capacity of Vegetable category; NCD, Neurocognitive disorder.

To elucidate the psychometric features of component‐specific measures of CVFT, the correlation between CVFT performance and cognitive function demonstrated diverse patterns in the elderly with different cognitive statuses. In high‐performing elderly, higher executive function (measured by TMT‐B) was correlated with better performance of CVFT 60 s (capacity‐based measure: *r* = −0.301, *p* = 0.029; speed‐based measure: *r* = 0.321, *p* = 0.019) (Table A4). In normal ageing elderly, higher executive function (measured by TMT‐B) was correlated with better performance of CVFT 30 s (capacity‐based measure: *r* = −0.201, *p* = 0.004; speed‐based measure: *r* = 0.237, *p* = 0.001) (Table A5).

In NCD patients, better performance of CVFT 30 seconds was correlated with higher global cognition (measured by HK MoCA) (capacity‐based measure: *r* = 0.247, *p* = 0.001; speed‐based measure: *r* = −0.255, *p* < 0.001) and attention (measured by DSF) (capacity‐based measure: *r* = 0.285, *p* < 0.001; speed‐based measure: *r* = −0.267, *p* < 0.001). Better performance of CVFT 60 seconds was correlated with higher global cognition (measured by HK MoCA) (speed‐based measure: *r* = −0.198, *p* = 0.006) and working memory capacity (capacity‐based measure: *r* = 0.205, *p* = 0.004; speed‐based measure: *r* = −0.188, *p* = 0.009) (Table A6).

### 3.2 ROC analysis

To classify the individuals with different cognitive statuses, the values of the area under the ROC curve (AUC) were used to test the discriminative power of component‐specific CVFT performance. ROC analysis showed that traditional measures as CVFT 30 s and CVFT 60 s had modest power to differentiate high‐performing elderly from normal ageing ones (CVFT 30 s: AUC value = 0.759, *p* < 0.001; CVFT 60 s: AUC value = 0.74, *p* < 0.001), and higher power to differentiate NCD patients from high‐performing elderly (CVFT 30 s: AUC value = 0.901, *p* < 0.001; CVFT 60 s: AUC value = 0.915, *p* < 0.001). Compared with conventional measures of CVFT, capacity of CVFT had similar differential value to differentiate normal ageing elderly and NCD patients (AUC value = 0.828, *p* < 0.001), but showed higher differential value than CVFT 30 s in the differentiation of normal ageing elderly from NCD patients (Capacity of CVFT: AUC value = 0.741, *p* < 0.001; CVFT 30 s: AUC value = 0.722, *p* < 0.001).

### 3.3 Morphometric correlates of capacity‐based measures

**FIGURE 2:**
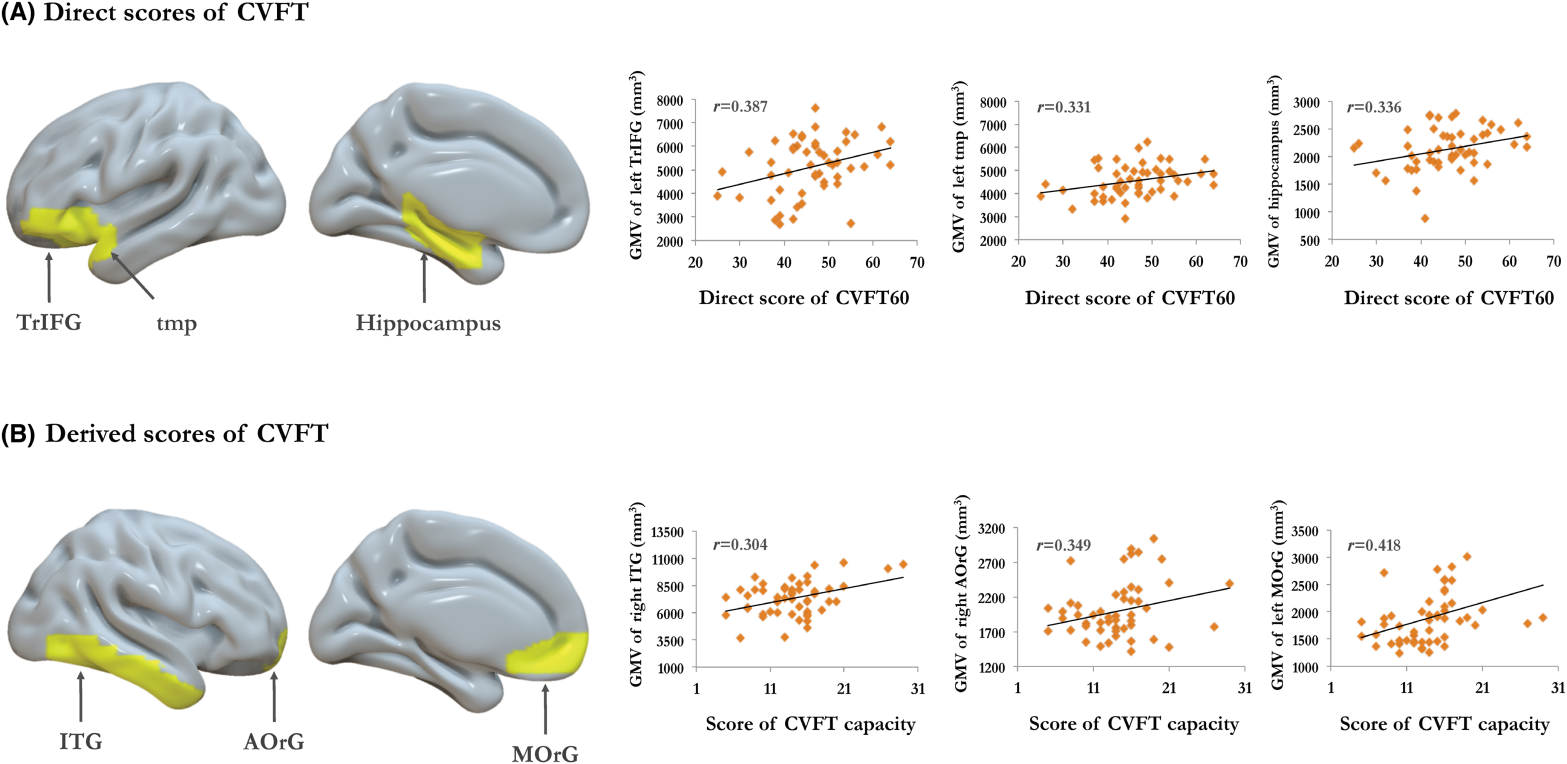
Morphometric correlates of capacity‐based measures of CVFT performance. The direct scores were positively correlated with the gray matter volume of TrIFG, tmp, and hippocampus (A). The derived scores were positively correlated with the gray matter volume of ITG, AOrG, and MOrG (B). Abbreviations: CVFT, Category verbal fluency test; ITG, Inferior temporal gyrus; LiG, Lingual gyrus; tmp, temporal pole; TrIFG, Inferior frontal gyrus‐pars triangularis.

Using age, gender, years of education, and total intracranial volume as covariates, direct scores of CVFT were correlated with left temporal pole (tmp) (*r* = 0.331, *p* = 0.02), left inferior frontal gyrus (IFG) (*r* = 0.387, *p* = 0.006), and left hippocampus (*r* = 0.336, *p* = 0.018) (Figure 2A). The derived scores of CVFT were correlated with left middle orbito‐frontal gyrus (*r* = 0.418, *p* = 0.003), right anterior orbito‐frontal gyrus (AOrG) (*r* = 0.349, *p* = 0.014), and right inferior temporal gyrus (*r* = 0.304, *p* = 0.034) (Figure 2B).

### 3.4 Morphometric correlates of speed‐based measures

**FIGURE 3:**
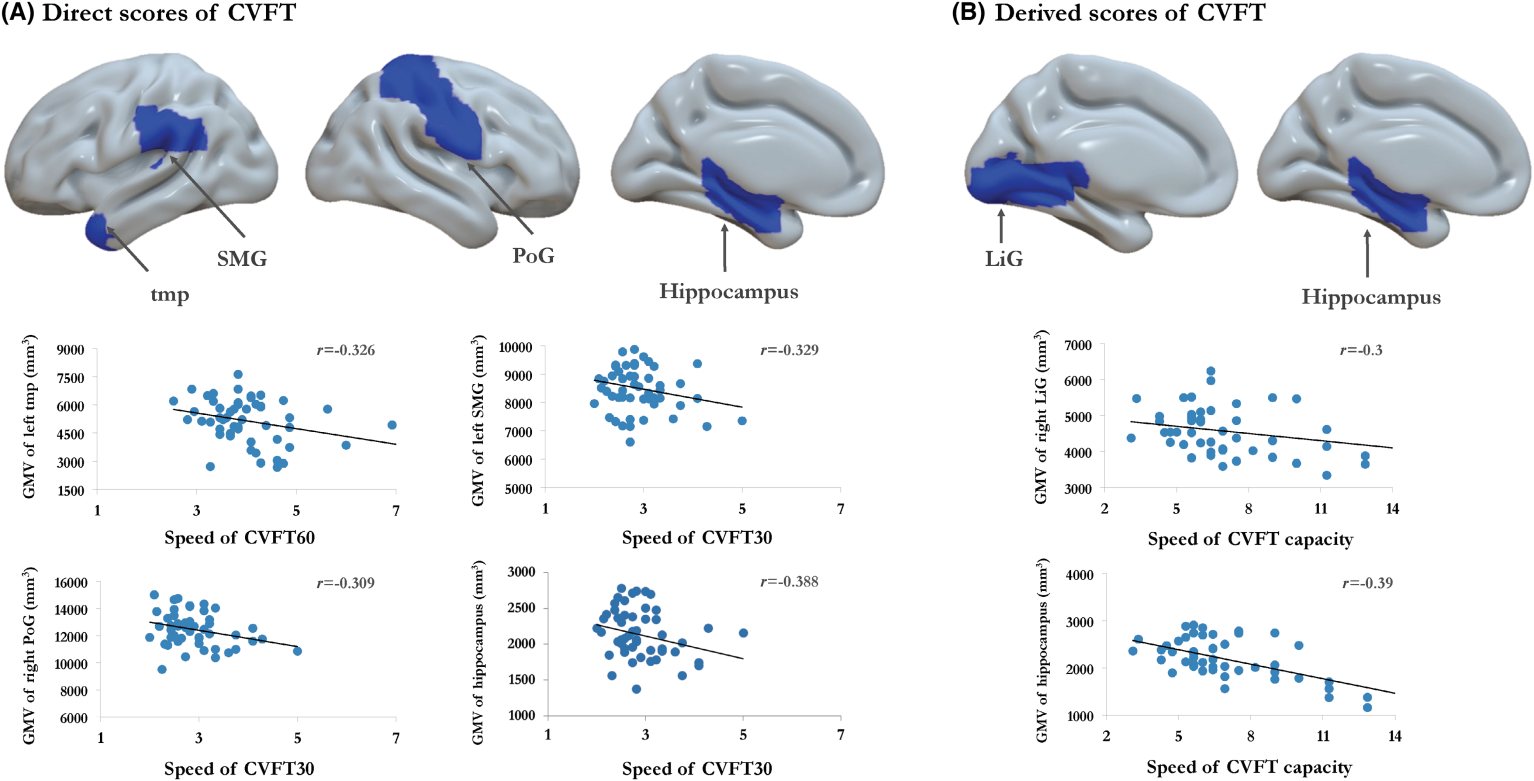
Morphometric correlates of speed‐based measures of CVFT performance. The direct scores were negatively correlated with the gray matter volume of PoG, SMG, tmp, and hippocampus (A). The derived scores were negatively correlated with the gray matter volume of hippocampus and LiG (B). Abbreviations: CVFT, Category verbal fluency test; LiG, Lingual gyrus; PoG, Postcentral gyrus; SMG, Supramarginal gyrus; tmp, temporal pole.

Slower speed of CVFT 30 seconds and CVFT 60 s was correlated with reduced GMV of left tmp (*r* = −0.326, *p* = 0.022), left supra marginal gyrus (*r* = −0.329, *p* = 0.021), right post‐central gyrus (PoG) (*r* = −0.309, *p* = 0.031), and left hippocampus (*r* = −0.388, *p* = 0.006) (Figure 3A). Speed of CVFT capacity was correlated with reduced left lingual gyrus (LiG) (*r* = −0.3, *p* = 0.036) and hippocampus (*r* = −0.39, *p* = 0.006) (Figure 3B).

### 3.5 Hemispheric lateralization of CVFT performance

With regard to the lateralized features, CVFT capacity was strongly correlated with the LI of AOrG (*r* = −0.411, *p* = 0.004) and the LI of LiG (*r* = 0.334, *p* = 0.02). Speed of CVFT was correlated with the LI of tmp (*r* = −0.327, *p* = 0.023). Speed of CVFT capacity was correlated with the LI of AOrG (*r* = 0.359, *p* = 0.012), but negatively related to the LI of LiG (*r* = −0.299, *p* = 0.039).

### 3.6 Brain age and BrainAGE

**FIGURE 4:**
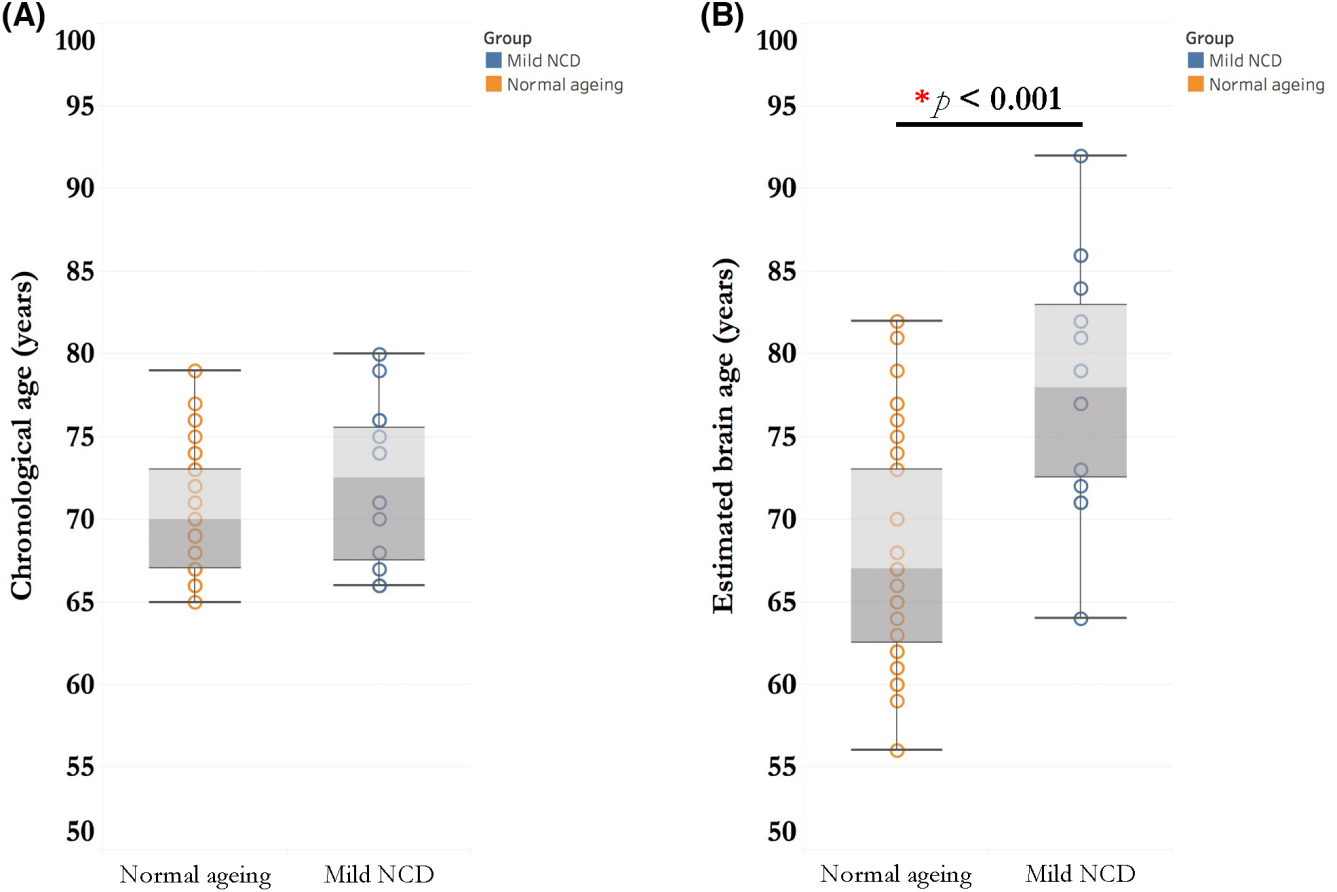
Comparisons of chronological age and estimated brain age in normal ageing elderly and mild neurocognitive disorder (NCD) patients. (A) There was no difference of chronological age between normal ageing elderly and mild NCD patients. (B) Mild NCD patients had older brain age than chronological age‐matched normal ageing elderly.

The mean absolute error of pre‐trained brain age model was 3.581, which had comparable generalizability with the published brain age models^44^, ^45^ and the testing dataset. Generally, the chronological age was positively correlated with estimated brain age in training samples (*r* = 0.737, *p* < 0.001) and our clinical samples (normal ageing elderly: *r* = 0.621, *p* < 0.001; mild NCD patients: *r* = 0.844, *p* < 0.001). As shown in Table 3, the demographics were comparable between normal ageing elderly and mild NCD patients. Mild NCD patients have worse global cognition and executive function than normal aging elderly. Although mild NCD patients had similar chronological age (*t* = −1.253, *p* = 0.216), pronounced increased brain age (*t* = −4.811, *p* < 0.001) and higher BrainAGE score (*t* = −5.637, *p* < 0.001) were found in mild NCD patients (Figure 4).

**TABLE 3:** Baseline demographics, clinical and brain features in normal aging and mild NCD groups.

Clinical features	Normal ageing (*n* = 36)	Mild NCD (*n* = 16)	*t* Value (*χ* ^2^)	*p* Value
Age	70.54 ± 3.65	72.04 ± 4.62	−1.253	0.216
Sex (F/M)	23/13	10/6	2.002	0.059
Education (years)	8.83 ± 4.81	8.72 ± 3.04	0.087	0.931
CSDD	0.28 ± 1.11	0.38 ± 0.89	−0.309	0.759
PSQI	5.33 ± 3.58	5.38 ± 4.27	−0.036	0.971
ADL	0.99 ± 0.01	0.98 ± 0.04	2.082	0.042
CDR‐SOB	0.25 ± 0.41	0.59 ± 0.45	−2.718	0.009
CMMSE	28.31 ± 1.28	27.51 ± 1.55	1.959	0.056
HK MoCA	27.61 ± 1.55	25.19 ± 2.26	4.495	<0.001
DSF	7.69 ± 0.89	7.06 ± 1.56	1.851	0.071
DSB	3.69 ± 1.35	2.94 ± 0.68	2.121	0.039
TMT‐A	12.49 ± 6.47	15.25 ± 7.18	−1.375	0.175
TMT‐B	67.27 ± 46.73	83.16 ± 41.91	−1.166	0.249
Brain age	68.11 ± 6.62	77.91 ± 7.13	−4.811	<0.001
BrainAGE	−2.43 ± 5.21	5.86 ± 4.07	−6.202	<0.001

*Note*: Data are raw scores and presented as mean ± SD.

Abbreviations: ADL, Activity of daily living scale; BrainAGE, Brain age gap estimation; CDR‐SOB, Clinical dementia rating‐sum of box; CSDD, The Cornell Scale for Depression in Dementia; DSB, Digit span backward; DSF, Digit span forward; NCD, Neurocognitive disorder; PSQI, Pittsburgh Sleep Quality Index; TMT‐A, Trail making test‐part A; TMT‐B, Trail making test‐part B.

### 3.7 Correlation analyses

**FIGURE 5:**
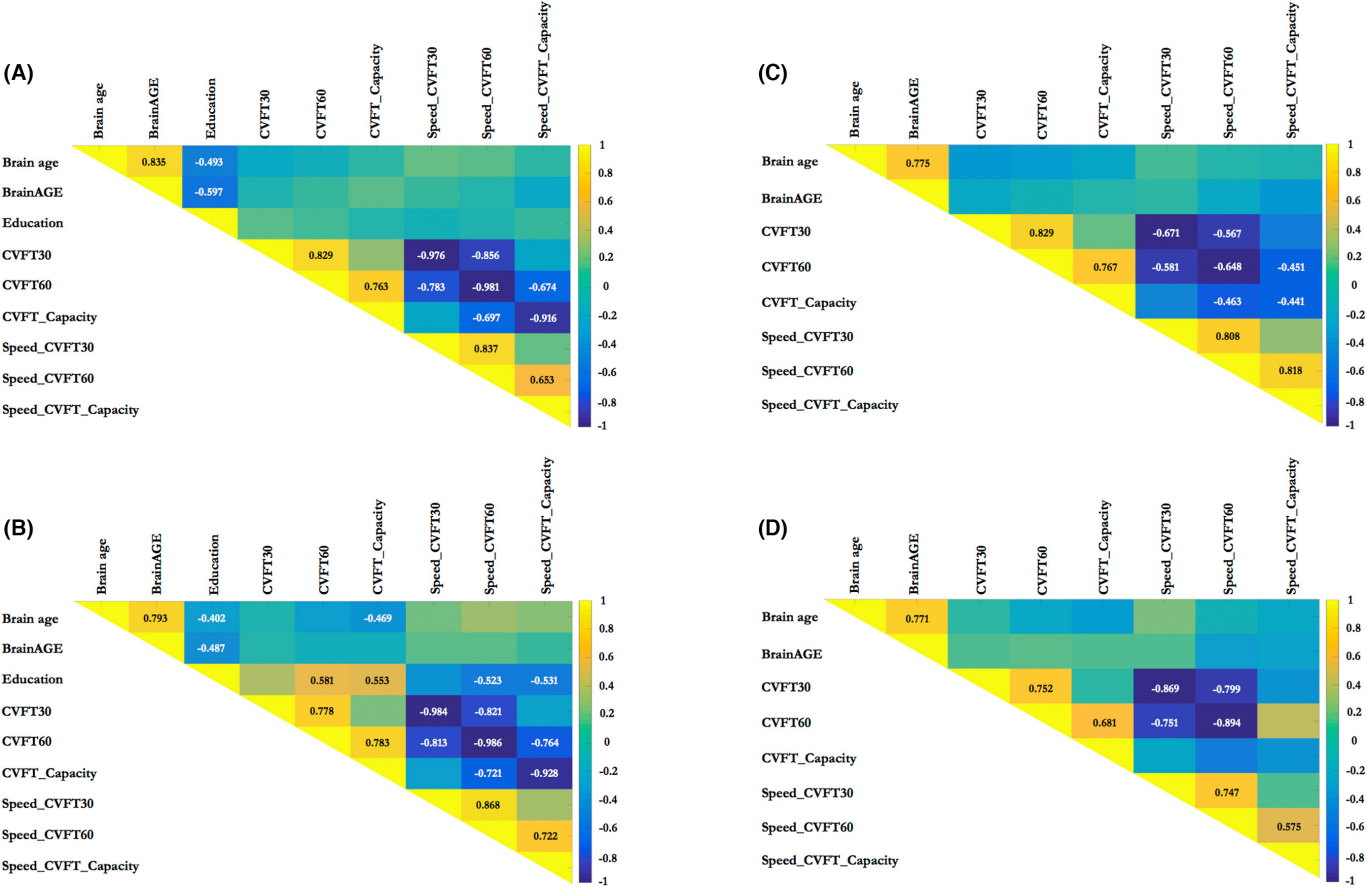
Correlation matrix for the category verbal fluency test (CVFT) performance and brain age matrices. Raw correlation matrix in normal ageing (A) and mild neurocognitive disorder (NCD) patients (B) and correlation matrix adjusted with the effects of education in normal ageing (C) and mild NCD patients (D). The color scale depicts the strength of the Pearson correlation coefficient. Superimposed text represents the actual numerical value of the Pearson correlation coefficient. Abbreviation: BrainAGE, Brain Age Gap Estimation.

To gain a better understanding of the relationships between brain age matrices, education, and the proxies of CVFT performance, correlation matrix was calculated in normal ageing and NCD groups. Overall, using age and gender as covariates, older estimated brain age was correlated with greater brain‐chronological age gap (i.e., BrainAGE) and less years of education. Capacity‐based measures of CVFT performance were significantly correlated with speed‐based measures in both groups. The Pearson correlation coefficients in normal aging group were lower than the ones in mild NCD group. Significant correlations between CVFT performance, brain age, and years of education were only found in mild NCD group (Figure 5B), not in normal ageing group (Figure 5A). In mild NCD patients, increased CVFT capacity was related to younger estimated brain age (*r* = −0.469, *p* = 0.034) and higher educational level (*r* = 0.553, *p* = 0.026). When adjusting the effects of education, the association between CVFT measures and brain age was unchanged in normal ageing group (Figure 5C), but the association between CVFT capacity and estimated brain age became non‐significant in mild NCD patients (*r* = −0.275, *p* = 0.259) (Figure 5D).

## 4 DISCUSSION

To the best of our knowledge, this study was the first to investigate the component‐specific CVFT performance and its associations with MRI‐informed brain age matrices in senior adults with normal ageing and mild neurocognitive disorder. Given the psychometric and morphometric features of CVFT, we found the task‐impurity problem in verbal fluency performance, indicating that CVFT has multiple components, including executive function, language, verbal storage, and working memory. Based on the component‐specific proxies, the individuals with different cognitive statuses exhibited the diversity in verbal fluency performance with corresponding cognitive and neural underpinnings. Not surprisingly, high‐performing elderly showed higher scores of CVFT than the ones with normal ageing and NCD patients; and the major NCD patients demonstrated the worse CVFT performance. Apart from the conventional proxies (i.e., CVFT 30 and CVFT 60), the component‐specific proxies, including capacity‐ and speed‐based measures, were developed to tackle the task‐impurity problem of CVFT performance.

Component‐specific decoding of verbal fluency performance is an analytic approach facilitated with psychometric features of CVFT. For instance, the capacity‐based measures presented extensive correlations with global cognition and attention in NCD patients. In contrast, the speed‐based measures had more specific associations with executive function across the groups with different cognitive statuses. The discrepancy of the “CVFT‐cognition” correlations might reflect the alternative dynamics of brain function in clinical populations, which have been well investigated throughout the course of disease progression. For example, compared to the patients with MCI, mild AD patients showed similar performance of memory, but a dramatic reduced number of word generation (i.e., capacity) during verbal fluency test.^8^, ^9^, ^10^, ^46^, ^47^ On the other hand, capacity‐based performance of CVFT might remain intact during healthy ageing, but speed‐based measures are disproportionately disrupted relatively to young adults.^48^ Of note, speed‐based measures of CVFT were strongly correlated with the scores of psychological tests than the capacity‐based measures of CVFT. The domain‐specific correlated patterns were also observed and confirmed in our morphometry study. Overlapped cortical regions, such as temporal pole and hippocampus, were found between component‐specific proxies and cortical volumes, whereas there are also some unique links, indicating the multiple components of CVFT as well.

Regarding to the long history of verbal fluency research, neuroimaging plays a vital role to link CVFT performance to specific brain features. For example, the first positron emission tomography study found an increased glucose metabolic rate in frontal and temporal lobes during CVFT.^49^ Functional MRI studies found a left lateralized CVFT‐related cortical activation pattern.^50^ Later, evidence from functional near‐infrared spectroscopy and electroencephalography also demonstrated significant lateralization during semantic verbal fluency test, particularly in PoG (right > left), hippocampus (left > right), and inferior frontal gyrus (left > right).^51^, ^52^ Indeed, the process of word production exhibits a hemispheric specialization. Consistent with previous studies,^53^ most of the CVFT measures showed a feature of left dominance (i.e., hippocampus, frontal gyrus, and lingual gyrus). Of note, when linking the lateralized features with CVFT performance, capacity‐based measures showed a left lateralized feature, whereas speed‐based measures showed a right lateralized feature.

Beyond the lateralized features of CVFT measures, another interesting finding is the associations between CVFT capacity and MRI‐informed estimated brain age in mild NCD patients. Based on the features of cortical GMVs, the estimated brain age represents the status of brain ageing at individual level.^40^, ^41^, ^42^ At present, although there is no direct link between brain age and executive function, the close relationship between age‐related brain atrophy (i.e., decreased brain reserve) and executive dysfunction suggests that the accelerated brain changes have significant effects on frontal functions in late adulthood.^54^ Not limited to executive function, we observed the diverse changes of verbal fluency performance across three categories showed greater discriminative value in NCD patients than in normal ageing elderly. Interestingly, the positive correlation between CVFT capacity and working memory capacity is more pronounced in NCD patients rather than high‐performing and normal ageing elderly, indicating that the impaired capacity related to accelerated brain ageing may be a fundamental change in early‐stage neurodegeneration.

To conclude, this study endorses the phenomenon that the deficits in verbal fluency performance are most frequently noticed cognitive dysfunction in normal ageing and NCD patients. The multiple components of CVFT, related to language, memory, and executive function, could be assessed using direct and derived proxies that have the relevant neural underpinnings. Therefore, the findings might deepen our understanding of verbal fluency performance characterized with component‐specific and lateralized function, and further help us create more specialized treatments for people who are at high risk of developing dementia.

### Limitations and future directions

Although the findings in this study are encouraging, conclusions need to be interpreted with caution due to its limitations. The educational levels across the four groups were not comparable. Considering both educational attainment and language ability (i.e., bilingualism) are key components of cognitive reserve,^55^, ^56^, ^57^ these variables might contribute to the group‐wise differences of CVFT performance and brain age matrices. Regarding the role of education in neurodegeneration, we adjusted effects of education on the correlation matrix of CVFT performance and brain age matrices and found the previous significant association between CVFT capacity and brain age disappeared. Besides, a relatively small collection of MRI scans might limit the generalization of the “CVFT‐brain” correlation patterns. Moreover, the absence of the status of language ability (i.e., bilingualism) on component‐specific CVFT performance may also limit our interpretations in the relationships of verbal fluency performance, estimated brain age, and cognitive reserve.

Despite the limitations, this study has several strengths. First, through decoding the complexity of CVFT performance (i.e., verbal and fluid components), the findings of this study may illustrate the importance of considering CVFT as an efficient screening tool for age‐related neurodegenerative diseases, particularly for vascular cognitive impairment, vascular dementia, and PD. Meanwhile, more sophisticated methods, such as syntactic analysis and structural analysis of CVFT performance, should be addressed in different types of brain disorders in future studies.

## AUTHOR CONTRIBUTIONS

Hanna Lu and Linda CW Lam conceived and designed this study. Jing Li and Hanna Lu calculated the brain age matrices. Ada WT Fung conducted the CVFT. As to this paper, Hanna Lu drafted, Ada WT Fung, and Linda CW Lam discussed and agreed with the final version.

## CONFLICT OF INTEREST STATEMENT

The authors declared no potential conflicts of interest with respect to the research, authorship, and/or publication of this article.

**TABLE A1:** Comparisons of CVFT performance between high‐performing and normal ageing elderly.

	High performing (*n* = 56)	Normal ageing (*n* = 205)	*F* value	*p* Value
Capacity‐based measures				
CVFT‐A30	14.71 ± 2.96	12.96 ± 2.91	3.992	<0.001
CVFT‐A60	20.73 ± 4.11	18.66 ± 4.35	3.191	<0.001
CVFT‐F30	12.14 ± 2.35	10.17 ± 2.22	5.825	<0.001
CVFT‐F60	16.21 ± 3.37	13.84 ± 2.95	5.181	<0.001
CVFT‐V30	11.93 ± 2.54	10.24 ± 2.61	4.311	<0.001
CVFT‐V60	18.29 ± 4.28	15.57 ± 4.04	4.411	<0.001
CVFT‐CA	6.02 ± 2.76	5.71 ± 2.81	0.736	0.463
CVFT‐CF	4.07 ± 2.28	3.67 ± 1.84	1.378	0.169
CVFT‐CV	6.36 ± 2.71	5.32 ± 2.44	2.744	0.006
Speed‐based measures				
CVFT‐A30	2.12 ± 0.42	2.44 ± 0.64	−3.613	<0.001
CVFT‐A60	3.01 ± 0.59	3.38 ± 0.77	−3.406	<0.001
CVFT‐F30	2.56 ± 0.49	3.11 ± 0.73	−5.173	<0.001
CVFT‐F60	3.87 ± 0.86	4.54 ± 1.04	−4.436	<0.001
CVFT‐V30	2.65 ± 0.71	3.14 ± 0.92	−3.698	<0.001
CVFT‐V60	3.47 ± 0.84	4.14 ± 1.18	−3.974	<0.001
CVFT‐CA	6.43 ± 4.29	6.91 ± 4.83	−0.668	0.505
CVFT‐CF	10.59 ± 8.28	10.37 ± 6.82	0.213	0.831
CVFT‐CV	5.34 ± 2.35	7.47 ± 5.89	−2.645	0.009

*Note*: Data are raw scores and presented as mean ± SD.

Abbreviations: A30, Animal category at 30 s; A60, Animal category at 60 s; CVFT, Category Verbal fluency Test; CVFT‐CA, Category Verbal fluency test‐Capacity of Animal category; CVFT‐CF, Category Verbal fluency test‐Capacity of Fruit category; CVFT‐CV, Category Verbal fluency test‐Capacity of Vegetable category.

**TABLE A2:** Comparisons of CVFT performance between normal ageing and mild NCD patients.

	Normal ageing (*n* = 205)	Minor NCD (*n* = 204)	*F* value	*p* Value
Capacity‐based measures				
CVFT‐A30	12.96 ± 2.91	11.07 ± 2.93	6.059	<0.001
CVFT‐A60	18.66 ± 4.35	15.31 ± 3.88	8.208	<0.001
CVFT‐F30	10.17 ± 2.22	8.91 ± 2.24	5.757	<0.001
CVFT‐F60	13.84 ± 2.95	11.69 ± 2.81	7.531	<0.001
CVFT‐V30	10.24 ± 2.61	8.95 ± 2.68	4.942	<0.001
CVFT‐V60	15.57 ± 4.04	12.88 ± 4.03	6.734	<0.001
CVFT‐CA	5.71 ± 2.81	4.19 ± 2.19	6.089	<0.001
CVFT‐CF	3.67 ± 1.84	2.79 ± 1.64	5.072	<0.001
CVFT‐CV	5.32 ± 2.44	3.93 ± 2.28	5.969	<0.001
Speed‐based measures				
CVFT‐A30	2.44 ± 0.64	2.89 ± 0.84	−6.136	<0.001
CVFT‐A60	3.38 ± 0.77	4.21 ± 1.26	−8.002	<0.001
CVFT‐F30	3.11 ± 0.73	3.63 ± 1.09	−5.712	<0.001
CVFT‐F60	4.54 ± 1.04	5.46 ± 1.47	−7.311	<0.001
CVFT‐V30	3.14 ± 0.92	3.71 ± 1.34	−4.922	<0.001
CVFT‐V60	4.14 ± 1.18	5.16 ± 1.78	−6.829	<0.001
CVFT‐CA	6.91 ± 4.83	9.61 ± 7.03	−4.527	<0.001
CVFT‐CF	10.37 ± 6.82	12.84 ± 8.88	−3.154	0.009
CVFT‐CV	7.47 ± 5.89	10.88 ± 8.18	−4.822	<0.001

*Note*: Data are raw scores and presented as mean ± SD.

Abbreviations: A30, Animal category at 30 s; A60, Animal category at 60 s; CVFT, Category Verbal fluency Test; CVFT‐CA, Category Verbal fluency test‐Capacity of Animal category; CVFT‐CF, Category Verbal fluency test‐Capacity of Fruit category; CVFT‐CV, Category Verbal fluency test‐Capacity of Vegetable category.

**TABLE A3:** Comparisons of component‐specific CVFT performance between mild and major NCD patients.

	Minor NCD (*n* = 204)	Major NCD (*n* = 23)	*T* value	*p* Value
Capacity‐based measures				
CVFT‐A30	11.07 ± 2.93	8.04 ± 3.07	4.677	<0.001
CVFT‐A60	15.31 ± 3.88	11.22 ± 3.59	4.821	<0.001
CVFT‐F30	8.91 ± 2.24	6.65 ± 1.89	4.636	<0.001
CVFT‐F60	11.69 ± 2.81	8.91 ± 2.91	4.494	<0.001
CVFT‐V30	8.95 ± 2.68	6.65 ± 2.66	3.903	<0.001
CVFT‐V60	12.88 ± 4.03	9.17 ± 3.03	4.271	<0.001
CVFT‐CA	4.19 ± 2.19	3.17 ± 1.83	2.133	0.034
CVFT‐CF	2.79 ± 1.64	2.26 ± 1.68	1.471	0.532
CVFT‐CV	3.93 ± 2.28	2.52 ± 1.21	2.908	0.004
Speed‐based measures				
CVFT‐A30	2.89 ± 0.84	4.53 ± 2.36	−6.847	<0.001
CVFT‐A60	4.21 ± 1.26	6.07 ± 2.63	−5.805	<0.001
CVFT‐F30	3.63 ± 1.09	4.95 ± 1.74	−5.172	<0.001
CVFT‐F60	5.46 ± 1.47	7.49 ± 2.67	−5.664	<0.001
CVFT‐V30	3.71 ± 1.34	5.13 ± 1.75	−4.657	<0.001
CVFT‐V60	5.16 ± 1.78	7.25 ± 2.34	−5.154	<0.001
CVFT‐CA	9.61 ± 7.03	12.34 ± 6.73	−1.771	0.078
CVFT‐CF	12.84 ± 8.88	13.41 ± 9.36	−0.291	0.771
CVFT‐CV	10.88 ± 8.18	13.31 ± 7.66	−1.356	0.177

*Note*: Data are raw scores and presented as mean ± SD.

Abbreviations: A30, Animal category at 30 s; A60, Animal category at 60 s; CVFT, Category Verbal fluency Test; CVFT‐CA, Category Verbal fluency test‐Capacity of Animal category; CVFT‐CF, Category Verbal fluency test‐Capacity of Fruit category; CVFT‐CV, Category Verbal fluency test‐Capacity of Vegetable category; NCD, Neurocognitive disorder.

**TABLE A4:** Correlation matrix between CVFT measures and cognitive function in high‐performing elderly.

	CVFT measures						Cognitive funtion									
	CVFT30	CVFT60	CVFT_Cap	SOC30	SOC60	SOC_Cap	CDR‐SOB	CMMSE	MoCA	ADAS‐Cog	DR	TMT‐A	TMT‐B	DSF	DSB	WMC
CVFT30	–															
CVFT60	0.796^#^	–														
CVFT_Cap	0.233	0.774^#^	–													
SOC30	−0.978^#^	−0.781^#^	−0.231	–												
SOC60	−0.789^#^	−0.982^#^	−0.752^#^	0.801^#^	–											
SOC_Cap	−0.247	−0.742^#^	−0.933^#^	0.249	0.758^#^	–										
CDR‐SOB	0.197	0.263	0.216	−0.217	−0.271	−0.167	–									
CMMSE	−0.105	−0.301*	−0.374*	0.083	0.268	0.259	−0.196	–								
MoCA	0.146	0.046	−0.079	−0.175	−0.048	0.091	−0.162	−0.042	–							
ADAS‐Cog	0.029	−0.051	−0.111	−0.051	0.034	0.076	−0.152	0.005	−0.003	–						
DR	0.056	0.175	0.222	−0.003	−0.174	−0.256	0.146	−0.197	−0.049	−0.457^#^	–					
TMT‐A	−0.076	−0.047	0.004	0.055	0.054	0.037	0.244	−0.171	0.051	0.051	0.013	–				
TMT‐B	−0.243	−0.301*	−0.228	0.252	0.321*	0.253	0.155	0.074	−0.073	−0.032	−0.081	0.746^#^	–			
DSF	0.164	0.219	0.181	−0.139	−0.169	−0.073	0.173	0.007	−0.074	−0.058	−0.053	0.141	0.259	–		
DSB	−0.038	−0.004	0.033	0.001	−0.018	0.002	−0.002	0.003	−0.128	−0.091	−0.091	−0.118	0.106	0.214	–	
WMC	0.173	0.247	0.216	−0.143	0.224	−0.184	0.297*	−0.061	−0.071	−0.431^#^	0.425^#^	0.181	0.266	0.736^#^	0.195	–

*Note*: Data are Pearson correlation coefficients. *p < 0.05; ^#^ *p* < 0.001.

Abbreviations: ADAS‐Cog, The Alzheimer's Disease Assessment Scale‐Cognitive Subscale; CMMSE, Cantonese mini‐mental state examination; CVFT, category verbal fluency test; DR, delayed recall; DSF, digit span backward; DSF, digit span forward; MoCA, Montreal Cognitive Assessment; SOC, speed of CVFT; SOC‐Cap, speed of CVFT capacity; TMT‐A, trail making test part A; TMT‐B, trail making test part B; WMC, working memory capacity.

**TABLE A5:** Correlation matrix between CVFT measures and cognitive function in normal ageing.

	CVFT measures						Cognitive function									
	CVFT30	CVFT60	CVFT_Cap	SOC30	SOC60	SOC_Cap	CDR‐SOB	CMMSE	MoCA	ADAS‐Cog	DR	TMT‐A	TMT‐B	DSF	DSB	WMC
CVFT30	–															
CVFT60	0.843^#^	–														
CVFT_Cap	0.321^#^	0.781^#^	–													
SOC30	−0.973^#^	−0.797^#^	−0.271^#^	–												
SOC60	−0.823^#^	−0.965^#^	−0.741^#^	0.823^#^	–											
SOC_Cap	−0.254^#^	−0.681^#^	−0.902^#^	0.225^#^	0.707^#^	–										
CDR‐SOB	−0.057	−0.004	0.059	0.059	0.003	−0.066	–									
CMMSE	0.025	−0.004	−0.036	−0.017	0.001	0.027	−0.442^#^	–								
MoCA	0.107	0.109	0.068	−0.094	−0.097	−0.033	−0.291^#^	0.091	–							
ADAS‐Cog	−0.118	−0.131	−0.093	0.096	0.099	0.067	0.099	−0.038	−0.115	–						
DR	0.061	0.078	0.066	−0.028	−0.051	−0.062	−0.064	−0.047	0.089	−0.535^#^	–					
TMT‐A	−0.018	0.035	0.083	0.007	−0.072	−0.137	0.108	−0.001	−0.151	−0.058	0.095	–				
TMT‐B	−0.201*	−0.112	0.036	0.237^#^	0.104	−0.088	0.143	0.001	−0.084	−0.021	0.048	0.421^#^	–			
DSF	0.129	0.127	0.073	−0.118	−0.114	−0.081	−0.031	−0.035	0.102	−0.183*	0.062	0.003	−0.074	–		
DSB	0.058	0.085	0.082	−0.073	−0.111	−0.067	−0.063	0.055	0.089	−0.005	−0.024	0.102	−0.134	0.193*	–	
WMC	0.086	0.101	0.077	−0.069	−0.102	−0.129	−0.089	0.006	0.179*	−0.508^#^	0.462^#^	0.057	−0.051	0.722^#^	0.142*	–

*Note*: Data are Pearson correlation coefficients. *p < 0.05; ^#^ *p* < 0.001.

Abbreviations: ADAS‐Cog, The Alzheimer's Disease Assessment Scale‐Cognitive Subscale; CMMSE, Cantonese mini‐mental state examination; CVFT, category verbal fluency test; DR, delayed recall; DSF, digit span backward; DSF, digit span forward; MoCA, Montreal Cognitive Assessment; SOC, speed of CVFT; SOC‐Cap, speed of CVFT capacity; TMT‐A, trail making test part A; TMT‐B, trail making test part B; WMC, working memory capacity.

**TABLE A6:** Correlation matrix between CVFT performance and cognitive function in NCD patients.

	CVFT measures						Cognitive function									
	CVFT30	CVFT60	CVFT_Cap	SOC30	SOC60	SOC_Cap	CDR‐SOB	CMMSE	MoCA	ADAS‐Cog	DR	TMT‐A	TMT‐B	DSF	DSB	WMC
CVFT30	–															
CVFT60	0.889^#^	–														
CVFT_Cap	0.397^#^	0.773^#^	–													
SOC30	–0.955^#^	–0.848^#^	–0.376^#^	–												
SOC60	–0.853^#^	–0.951^#^	–0.725^#^	0.888^#^	–											
SOC_Cap	–0.256^#^	–0.492^#^	–0.633^#^	0.257^#^	0.566^#^	–										
CDR‐SOB	–0.187*	–0.141	–0.023	0.175*	0.142	0.054	–									
CMMSE	0.135	0.103	0.021	–0.117	–0.092	–0.086	–0.413^#^	–								
MoCA	0.247	0.184*	0.025	0.255^#^	–0.198*	–0.057	–0.445^#^	0.297^#^	–							
ADAS‐Cog	–0.175*	–0.152	–0.062	0.169*	0.156*	0.071	0.334^#^	–0.319^#^	–0.467^#^	–						
DR	0.092	0.106	0.084	–0.089	–0.097	–0.093	–0.155*	0.118	0.091	–0.462^#^	–					
TMT‐A	–0.239^#^	–0.181*	–0.032	0.202*	0.141	–0.029	0.135	–0.023	−0.071	0.046	−0.026	–				
TMT‐B	−0.185*	−0.102	0.053	0.188*	0.111	−0.042	0.181*	−0.073	−0.204*	0.079	−0.067	0.532^#^	–			
DSF	0.285^#^	0.173*	−0.047	−0.267^#^	−0.166*	−0.011	−0.085	0.104	0.205*	−0.107	0.031	−0.074	−0.063	–		
DSB	0.137	0.055	−0.081	−0.122	−0.064	0.031	−0.058	0.059	0.221*	−0.119	−0.041	0.014	−0.063	0.256^#^	–	
WMC	0.247^#^	0.205*	0.069	−0.221*	−0.188*	−0.089	−0.128	0.174*	0.275^#^	−0.467^#^	0.481^#^	−0.038	−0.058	0.681^#^	0.191*	–

*Note*: Data are Pearson correlation coefficients. *p < 0.05; ^#^ *p* < 0.001.

Abbreviations: ADAS‐Cog, The Alzheimer's Disease Assessment Scale‐Cognitive Subscale; CMMSE, Cantonese mini‐mental state examination; CVFT, category verbal fluency test; DR, delayed recall; DSF, digit span backward; DSF, digit span forward; MoCA, Montreal Cognitive Assessment; SOC, speed of CVFT; SOC‐Cap, speed of CVFT capacity; TMT‐A, trail making test part A; TMT‐B, trail making test part B; WMC, working memory capacity.

## Data Availability

The dataset used and analyzed during the present study are available from the corresponding author on reasonable quest.
